# A Case of Epithelioma

**Published:** 1888-10

**Authors:** J. B. Elliott

**Affiliations:** Brooklyn


### A CASE OF EPITHELIOMA.

This disease, of late years so much the subject of attention, is one which has come forward, in name at least, since my earlier years of study and is one which has seldom fallen under my personal observation. But a case recently came into my hands, which in its progress, treatment, and result, excited great anxiety, much study, and considerable disappointment.

The patient, J. W. E., of this city, in his seventy-second year, of excellent family, constitution, and general good health, never having had any continued illness, had been for some months annoyed with a small sore upon the upper and outer rim of the left ear, without recollection exactly how or when it came. He had tried in many ways, without consulting a physician, to heal it, principally by the use of such mild or soothing applications as were of note, and at one time before it came to my notice, had applied a strong solution of caustic potash, but without benefit or seeming temporary relief.

By accident only did it at last fall under my observation, for still expecting the sore to go, he preferred to hide it from notice by brushing his sidelocks over it. But one day as he was before me the raw, red spot, devoid of its usual covering, attracted my attention, and then it was upon inquiry discovered that the little spot, not more than a quarter of an inch in extent, had been in existence in some degree for nearly or quite a year. In appearance it was simply an ordinary ulcerative sore surrounded by healthy inflammation. Except from its obstinacy in healing, as learned in the account given of it by the patient, no difficulty was anticipated in restoring the ear by ordinary methods.

And so with considerable confidence and without difficulty the opposing raw surfaces of the lesion were drawn and held together by properly adjusted adhesive straps. The immediate effect of this was encouraging, a seemingly healthy crust forming to unite and heal by the usual process. But about the third day this crust, like those which had preceded it over the former raw surface, began to lift and come away in flakes from a still moist, raw instead of a dry, healing surface underneath. Then new matter would again exude, dry, separate, and come away in this form every few days, but without progress in diminishing or healing the sore. In fact, after a time it became abundantly apparent that this treatment was useless if not actually injurious, and further attempts in this direction were abandoned.

Attention was next directed to constitutional instead of local treatment, and the use of remedies internally administered adopted. But here arose a formidable difficulty in selecting agents best calculated to meet this end. Under the law of similars it is very seldom that some tangible symptom is not present upon which we can build a fair prescription. But in this case from beginning to end there was positively nothing in the way of causes, symptoms, subjective or objective, or the sign of pathological condition outside of the little sore itself to guide the physician in the selection of remedies. It was simply a little sore upon the ear, but as time went on it proved not only obstinate but actually aggressive by constantly though very slowly advancing in size, and yet with no apparently disturbing effect upon the general system or continued good health of the patient. The little pain felt was entirely local, not extending in any direction, the glands of the neck were undisturbed by swelling or tenderness, all the organs of the body acted normally and well, sleep was undisturbed and good except that occasionally turning the head with the sore ear to the pillow would cause pain and sometimes awaken the patient. Thus, with no positive guide in the selection of a healing agent, resort was had to antipsoric and some of the so-called tissue remedies, hoping thereby for either direct benefit or the development of symptoms through which more exact treatment could be introduced. At first Silicea, one of the most charming medicines in affections of the cartilaginous and bony tissues, was administered and time given for it to act. Of course it was given in a high attenuation. Inert as a drug, its action in the two hundredth attenuation where plainly indicated, is the most positive of any curative agent that I have ever met. But though given in varied strengths it failed in this instance of any observable effect. In the meantime consultation was had with the best professional talent, and the use of several other seemingly well-selected remedies at fair intervals given a reasonable opportunity to act in the conduct of the case. Prominent among the remedies used were Arsenicum, Lachesis, Nit. acid, Psorinum, Thuja, Silicea, and Spongia; afterward the less used were Clematis, Creosotum, Croton tig., Graphites, Lycopodium, Sulphur, and Plumbum.

If any decisive effect was produced upon the lesion or upon the patient, even to the extent of a proving of any of these remedies, I failed to find it.

At one time Arsenicum, the most continued of any one medicine, was thought to be acting favorably, with some arrest of the slowly spreading sore, but as this did not continue it was abandoned, after reasonable waiting, and other remedies given.

But no progress in the right direction being found after a twelvemonth's treatment, but rather a slowly eating away of the ear, and yet so slow that at the end of this time the sore had gained hardly three-quarters of an inch in extent with a slight depth, it was thought best to consult surgical skill in the case. A surgeon in New York city of much distinction, the author of a valuable surgical work, and at the head of a popular clinique in the special treatment of epithelioma and other skin diseases in that city, with a reputation of knowing by constant familiarity and observation the best methods of procedure in such cases, was consulted and the patient presented to him for examination.

His immediate verdict was epithelioma, which could certainly be cured perfectly in a few weeks. Various plates, photographic and otherwise, representing the disease in various stages of development and cure were exhibited and favorably commented upon. The age of my patient and other important considerations were discussed, and a most favorable prognosis made as to results of the proposed treatment, which was urged to be at once adopted. An arsenical ointment applied directly upon the open sore was to do the work. The patient was told of the contemplated action, to which he most hopefully and cheerfully assented and an immediate preparation of the fresh arsenical mixture was made and applied. The patient went home suffering but in heroic good spirits. The next day I found him in great pain. His wife said he had passed a night of sleepless agony. The ointment had been removed from the ear at the end of sixteen hours from its application, as directed by the surgeon, and about six hours before I saw him. At this time the surface of the sore was swollen, frightfully inflamed, and fairly broiling with oozing matter. Through this day and another sleepless night this condition continued, though gradually subsiding, so that by the following day he was comparatively comfortable. In about ten days the inflammation had disappeared and a large dry, hard and healthy-looking scab adhered closely over most of the former raw surface. About this time the surgeon was again visited and everything pronounced favorably progressing as expected. In another week, when the patient called again, a small portion of the sore was still moist and oozing, to which a fresh application of the ointment was made. The same painful and other disturbing results followed as before though in a much less degree. After this a more comfortable state followed, the whole former open surface assumed a dry healing state, which continued right along steadily, so that by the end of the sixth week the dry scab separated and came away, leaving a perfectly healed surface underneath.

Now this phase of the case seemed exceedingly satisfactory.

I thanked the surgeon and congratulated the patient. But he did not respond to the congratulations as heartily as I expected, although still pleased and hopeful. In fact he did not feel well nor did he rally up to a good standard of vigor or strength. A somewhat laborious business in which he was engaged was resumed but with effort and less ability to continue exertion.

Presuming this state to be temporary only, the patient was allowed to disappear from frequent observation, but afterward as seen occasionally the ear was found all right. But the general appearance of the man was not satisfactory. He looked worn and felt tired. Appetite and digestion were sluggish and imperfect; sleep restless and unrefreshing; neuralgic pains in the head and elsewhere often troublesome.

The surgical treatment was resorted to in December, 1887, and the ear healed entirely in February, 1888, or in about six weeks, and two years or more after the sore was first noticed. He was now in his seventy-fourth year. From the time the ear healed all through the spring months the patient gradually failed and grew weaker. Shortness of breath became a troublesome though not a painful annoyance upon slight exertion. This, with other depressing conditions of body and mind, led the patient to call upon me early in June. Although he still kept at his business or its superintendence it was a most tiresome effort to go up-stairs or move hurriedly, and I was somewhat shocked when in this state he called upon me now for advice. On inquiry I found the difficulty of breathing was increased by lying down, and a restless condition with anxiety, a weakened, irregular action of the heart, dry mouth, thirst for cold drinks in small quantities, poor appetite, burning in chest, palpitation at night, urging to urinate with partial inability, weak feeling of head, face puffy and red as if feverish, mind sluggish and depressed as well as irritable, and other prominent symptoms of arsenical action as found recorded in our *Symptomen Codex.*

I prescribed Lachesis $^{200}$, and was to be seen or sent for if no improvement followed soon. In a few days a message came to call upon the patient, and with a brother physician the visit was made. It was now apparent that the heart's action was failing as well as intermittent and unsteady. Soon the patient was unable to lie down at all without a suffocative attack. All the organs except the heart performed their functions, though, of course, with diminished vital force. At length the heart itself ceased all regular or distinct beating and became as though a staying-pin had been removed with nothing left to steady or control its action. In its place was a tumbling tumult of labored movement with tired out distress of the patient, until he at last laid down exhausted, went into an unconscious state, and in twenty-four hours more had ceased to live. His death occurred from heart failure on the 17th of June, just about six months from the date of his first surgical treatment.

What caused this heart failure? I wrote to the surgeon who treated the case with a suggestion of an arsenical poisoning, which he would not admit, because death had not been a more immediate result of its application. His conclusion on this ground was not to me convincing or satisfactory. He suggested age as the probable cause, but this was discussed before the arsenic was applied when no sign of ill health or breaking down had occurred. Kidney trouble was also mentioned. The action of these organs had been carefully noted and urine tested with no sign of abnormal condition. The heart itself as a seat of primary trouble was, of course, thought of, but this, too, had acted steadily and perfectly while its sounds were clear and healthy up to the time of treatment mentioned.

There had been some catarrhal condition of the throat and nasal cavities, but of no unusual or important significance.

In view, then, of the general healthy organic condition and vigorous age preceding the surgical treatment of the patient in this case, how shall we account for the sudden and immediate breaking down and decline of the patient after that event? Is it at all probable that it would have occurred at this time without the interference of the operation? If not, then in what way was the operation injurious? Was it by the sudden suppression or shutting up in the system of the humor heretofore escaping through the sore, or the severe shock produced upon the patient by the direct violent action of the Arsenic, or upon this together with its secondary poisonous effect by absorption, or upon these various causes, together with the advanced age, unable to withstand such a formidable combination? In this connection comes up the interesting inquiry as to the fatality of this disease if left to itself, and the length of time a subject of it could live comfortably undisturbed by any treatment. The object of this paper will not be accomplished except by exciting attention, discussion, and comments upon these points and also as to the availability of medicinal treatment in this disease which if anywhere must be found in our school. Imperfect results or doing one thing at the expense of another in the treatment of patients will not satisfy.

It is one thing to suppress a sore and another, without injury, to heal the whole man.

BROOKLYN. J. B. ELLIOTT, M. D.

